# A High-Accuracy AlGaN/GaN Reverse Blocking CRD (RB-CRD) with Hybrid Trench Cathode

**DOI:** 10.1186/s11671-019-2860-y

**Published:** 2019-01-15

**Authors:** Anbang Zhang, Qi Zhou, Chao Yang, Yuanyuan Shi, Wanjun Chen, Zhaoji Li, Bo Zhang

**Affiliations:** School of Electronic Science and Engineering, State Key Laboratory of Electronic Thin Films and Integrated Devices, University of Electronic Science and Technology of China, Chengdu, 610054 China

**Keywords:** AlGaN/GaN heterostructure, Reverse blocking current regulating diode (RB-CRD), Schottky barrier diode (SBD)

## Abstract

An AlGaN/GaN lateral reverse blocking current regulating diode (RB-CRD) with trench Schottky anode and hybrid trench cathode has been proposed and experimentally demonstrated on silicon substrate. The Schottky barrier diode (SBD) integrated in the anode exhibits a turn-on voltage of 0.7 V and a reverse breakdown voltage of 260 V. The hybrid trench cathode acts as a CRD, which is in series connection with the anode SBD. A knee voltage of 1.3 V and a forward operation voltage beyond 200 V can be achieved for the RB-CRD. The RB-CRD is capable of outputting an excellent steady current in a wide temperature range from 25 to 300 °C. In addition, the forward regulating current exhibits small negative temperature coefficients less than − 0.152%/^o^C.

## Background

Wide bandgap semiconductors have attracted a considerable attention for the next generation of high-power, high-frequency, and high-temperature devices. GaN is one of the most promising wide bandgap semiconductors due to its superior properties such as large bandgap, high electron mobility, and high critical electric field [1–5]. In addition, due to the combination of spontaneous polarization and piezoelectric polarization, a high-density two-dimensional electron gas (2DEG) can be achieved at the AlGaN/GaN heterointerface. Such excellent properties enable the AlGaN/GaN-based power devices to operate with a low on-resistance while maintaining a high breakdown voltage. GaN-on silicon (GaN-on-Si) platform [6–8] has been regarded as the most promising technology towards high-performance and low-cost power devices, owing to the availability of large-diameter silicon wafers and the compatibility with the existing-matured CMOS fabrication process. Up to date, a variety of power devices [9–16] have been demonstrated on AlGaN/GaN-on-Si and some of them are commercially available. At the same time, the development of AlGaN/GaN device with new functionality may expand the application potential of AlGaN/GaN-on-Si, which is beneficial for boosting the extensive commercialization of AlGaN/GaN technology.

**Fig. 1:**
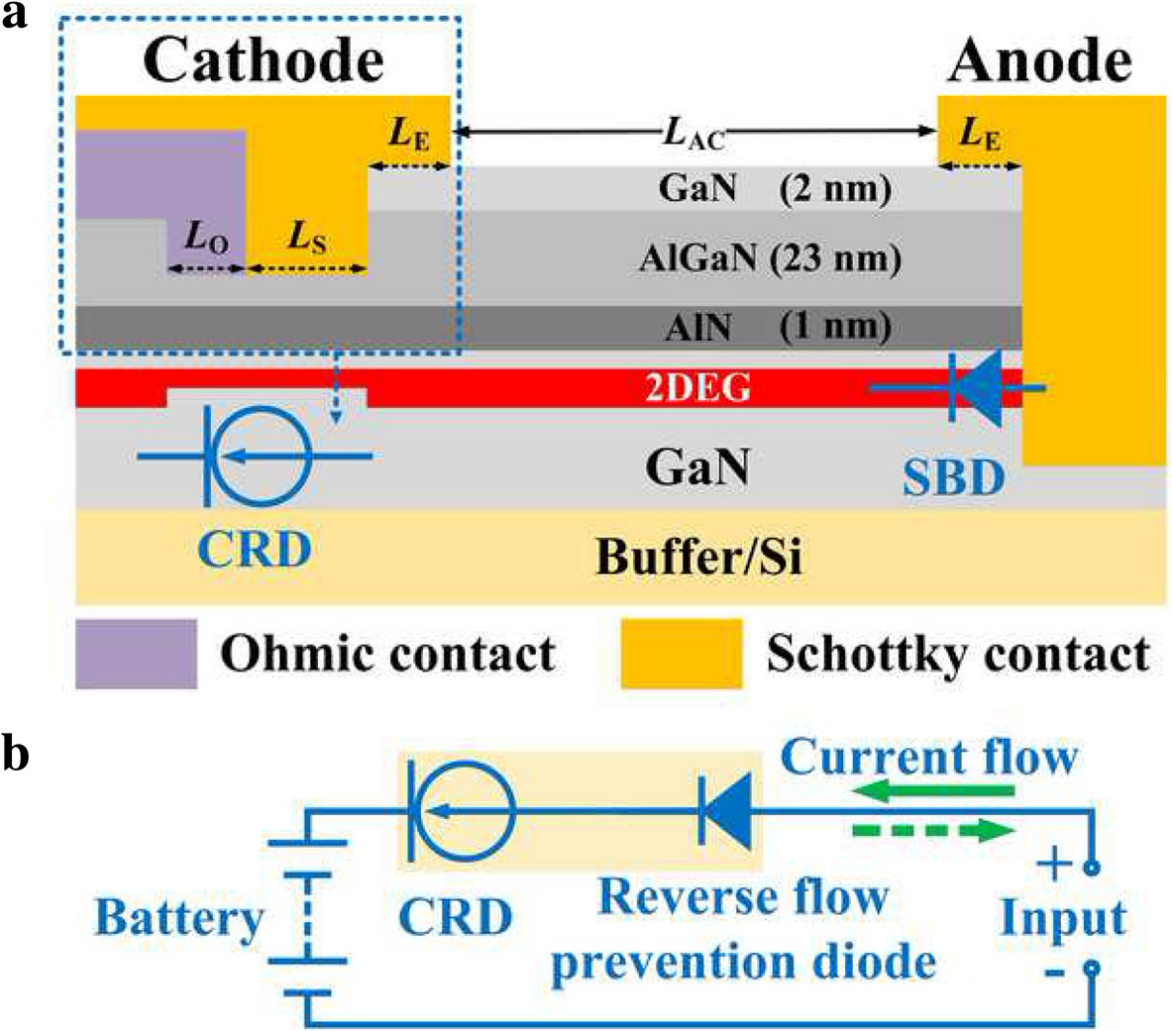
**a** Schematic cross section of the RB-CRD. **b** Circuit diagram of battery charging using the RB-CRD

As presented in Fig. 1a, in this work, a new type device termed as reverse blocking current regulating diode (RB-CRD) was experimentally demonstrated on AlGaN/GaN-on-Si. The RB-CRD features a trench Schottky anode and a hybrid trench cathode. A trench Schottky barrier diode (SBD) is formed at the anode while a CRD is achieved in the hybrid trench cathode. The RB-CRD can be regarded as a SBD in series connection with a CRD. A typical application of the RB-CRD is battery charging as shown in Fig. 1b. In the aforementioned battery charging circuit, the CRD acts as a constant current source, which output a constant current to charge the battery [17–19] regardless of the forward voltage fluctuation between the input and the battery. If the input voltage falls below the battery voltage, the reverse biased SBD in the circuit will prevent the battery from discharging.

## Methods

**Fig. 2:**
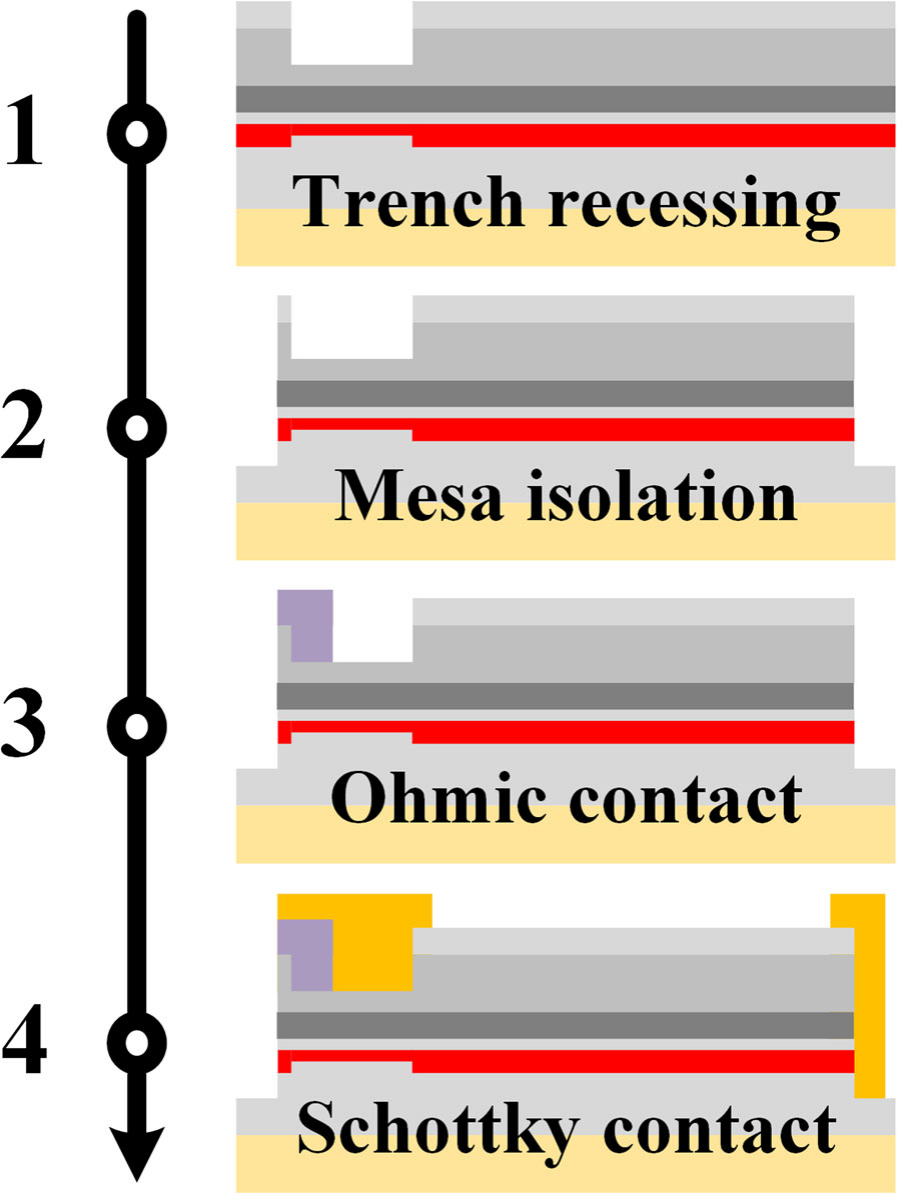
Manufacturing process flow of the RB-CRD

**Fig. 3:**
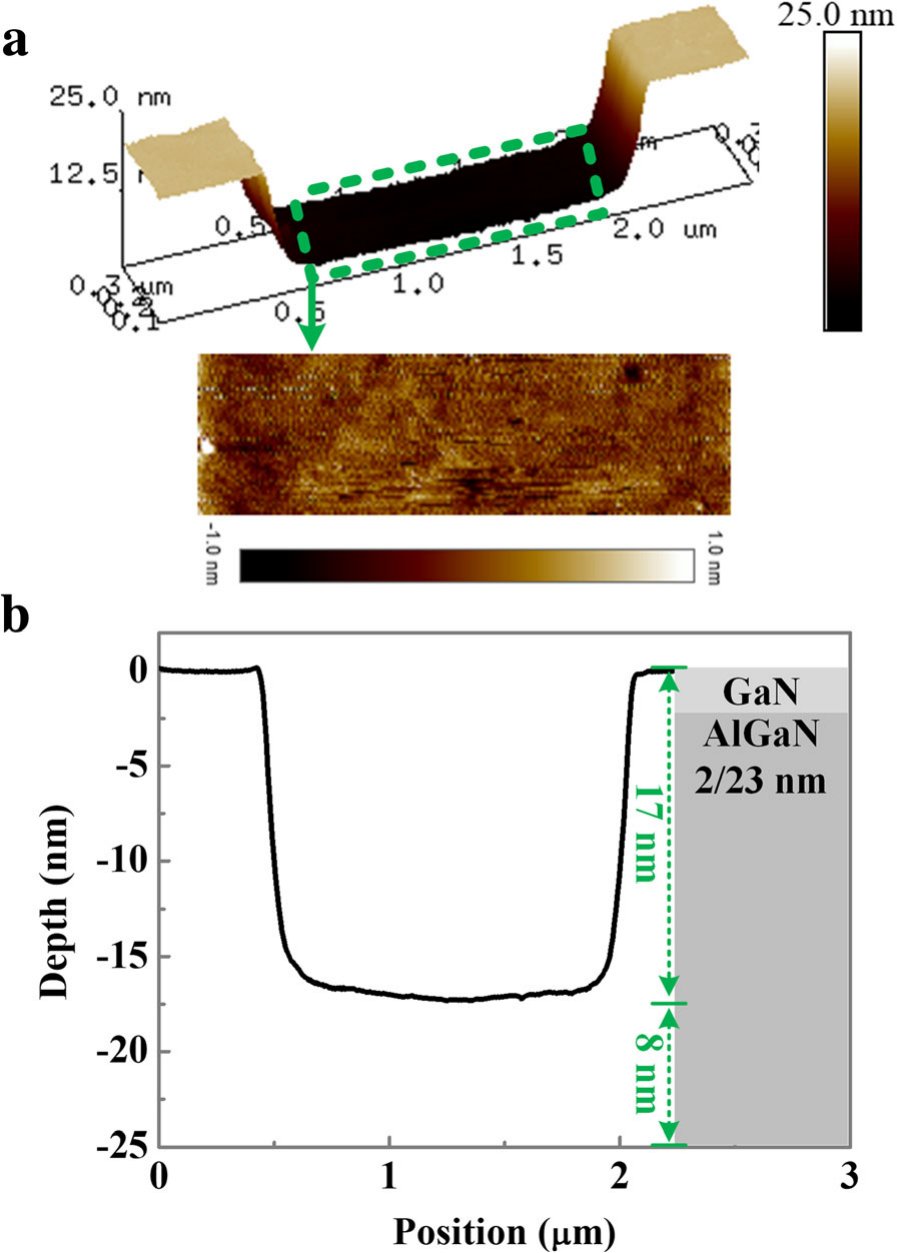
**a** AFM images of the cathode trench. **b** Height profile taken from the cathode trench

The epitaxial AlGaN/GaN heterostructure used for fabricating the RB-CRDs was grown on 6-in (111) silicon substrate by metal organic chemical vapor deposition (MOCVD). The epitaxial layers consist of 2-nm GaN cap, 23-nm AlGaN barrier, 1-nm AlN interlayer, 300-nm GaN channel, and 3.5-μm buffer. The Hall effect measured density and mobility of the 2DEG were 9.5 × 10^12^ cm^−2^ and 1500 cm^2^/V·s, respectively. The device fabrication process is shown in Fig. 2. First, a shallow trench (see Fig. 3) was etched in the cathode of the RB-CRD by a low power Cl_2_/BCl_3_-based inductively coupled plasma (ICP) etching technique. An etching rate of 7 nm/min was observed using the developed etching recipe with a RF power of 20 W, an ICP power of 60 W, a Cl_2_ flow of 5 sccm, and a BCl_3_ flow of 10 sccm. Then, mesa isolation with a depth of 300 nm was formed using the same ICP etching technique to disconnect the devices. The anode trench was accomplished by this process simultaneously. After that, the Ti/Al/Ni/Au (20/150/55/60 nm nm) metal stacks were deposited by the electron beam evaporation, followed by the rapid thermal annealing at 880 °C for 35 s in N_2_ ambient. The ohmic contact resistance of 1.1 Ω mm and sheet resistance of 400 Ω/square were extracted by the transmission line method. Finally, the device fabrication process ended up with the Ni/Au (50/300 nm) Schottky metal stack deposition. The distance between the anode and cathode (*L*_AC_) is 4 μm. The lengths of the ohmic contact (*L*_O_) and the Schottky contact (*L*_S_) in the cathode trench are 0.5 μm and 1 μm, respectively. The extended overhang (*L*_E_) of the Schottky contact is 0.5 μm.

## Results and Discussion

Figure 3a shows the 3D atomic force microscope (AFM) image of the fabricated cathode trench. The surface roughness of the bottom of the cathode trench is 0.3 nm. Such a small surface roughness is beneficial for the following metal-semiconductor contact. As shown in Fig. 3b, with a 17-nm depth cathode trench recessing, the 8-nm AlGaN barrier layer remains in the cathode trench region. Such a remaining AlGaN barrier layer enables that the 2DEG channel in the cathode trench region is always existing at zero bias.

**Fig. 4:**
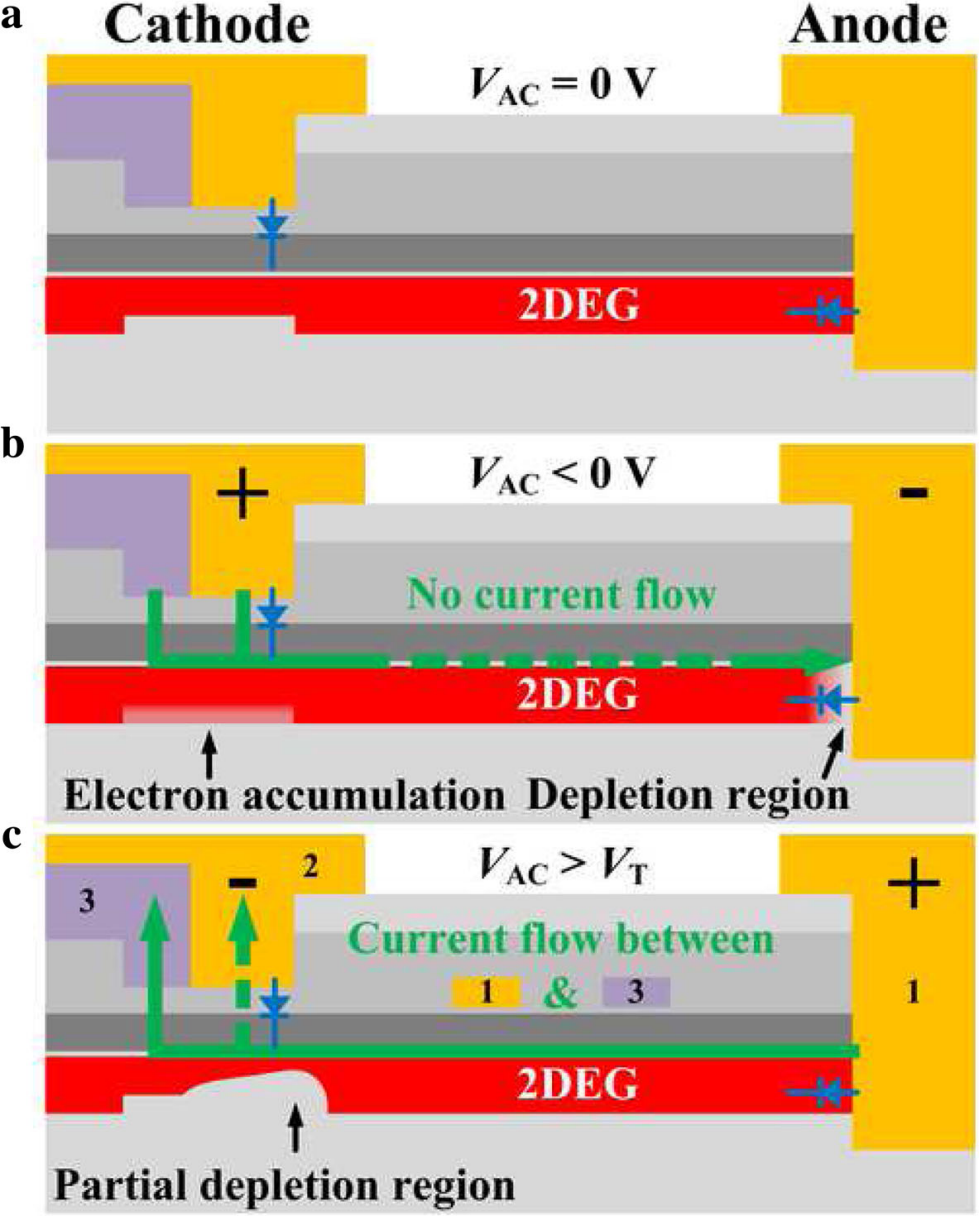
Schematic operation mechanism of the RB-CRD under **a** zero bias, **b** reverse bias, and **c** forward bias conditions

Figure 4 illustrates the operation mechanism of the RB-CRD. When a zero bias is applied to the anode (*V*_AC_ = 0 V) (see Fig. 4a), the RB-CRD is analogous to a Schottky-drain depletion-mode HEMT with the gate-source electrodes connecting. When a negative bias is applied to the anode (*V*_AC_ < 0 V) (see Fig. 4b), the electrons will accumulate in the cathode trench region while the 2DEG channel will be depleted in the anode region due to the reverse biased Schottky junction. There is no desired current following between the anode and the cathode, and the RB-CRD acts as a reverse biased SBD. As shown in Fig. 4c, when a positive bias which is beyond the turn-on voltage (*V*_*T*_, at 1 mA/mm) of the anode SBD is applied to the anode (*V*_AC_ > *V*_*T*_), the electrons will flow between the ohmic contact in the cathode and the Schottky contact in the anode. Meanwhile, the Schottky junction in the cathode is reverse biased and the 2DEG channel under the Schottky contact will be gradually depleted with increasing the forward bias. Therefore, the output current will initially increase with the applied anode voltage and then gradually reach saturation. In such case, a steady output current can be obtained.

**Fig. 5:**
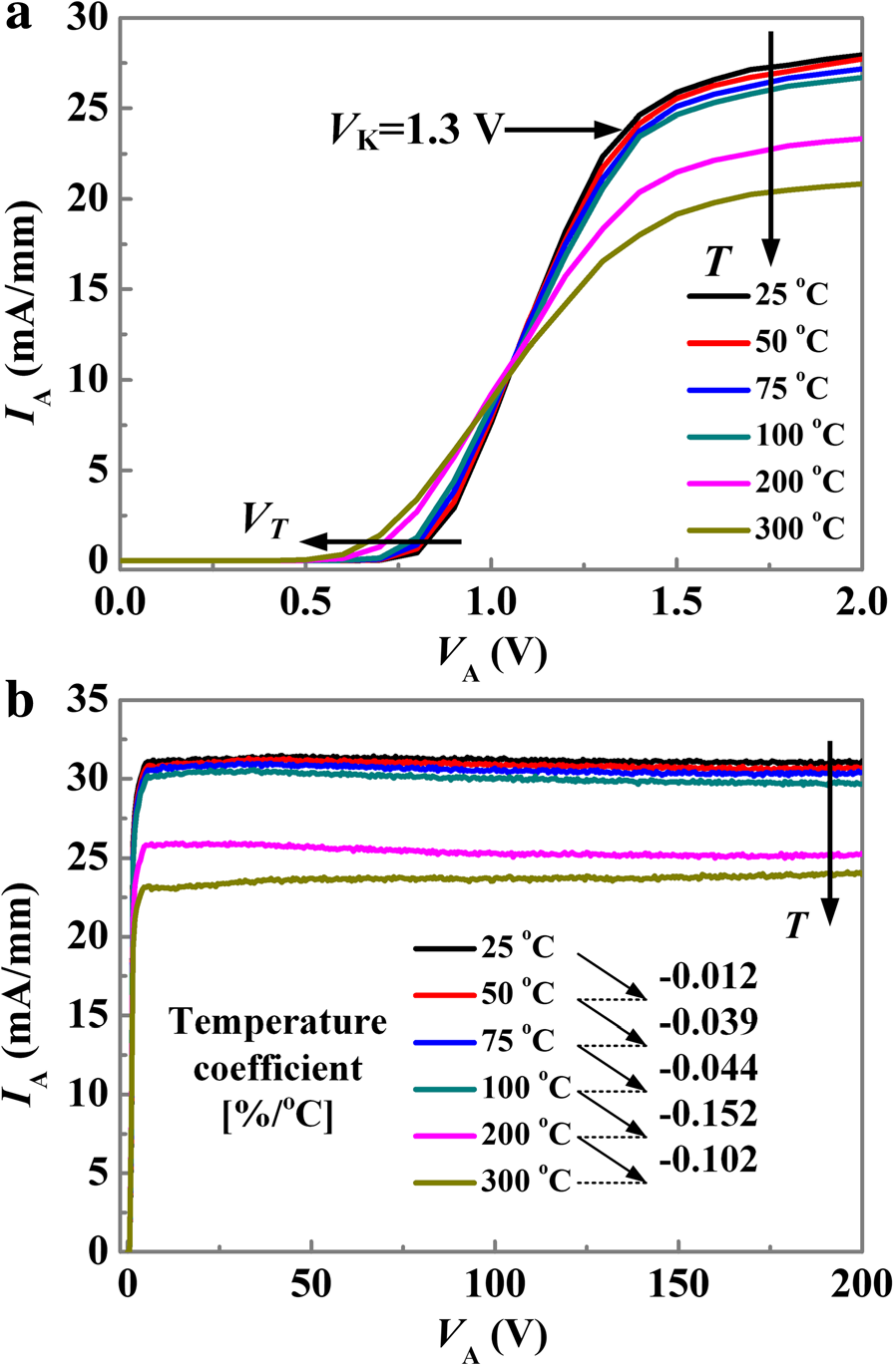
Temperature dependent forward bias *I*-*V* characteristics of the RB-CRD. Anode voltage ranges: **a** 0–2 V, **b** 0–200 V

The temperature-dependent forward *I-V* characteristics of the RB-CRD on the wafer are shown in Fig. 5. As shown in Fig. 5a, for the RB-CRD, a knee voltage (*V*_*K*_, at 80% of the steady regulating current) of 1.3 V is obtained which is higher than that of our previously reported CRDs (e.g., typical value 0.6 V) [20, 21]. This is due to the additional voltage drop (e.g., typical value 0.7 V) on the anode SBD of the RB-CRD. With the temperature increasing from 25 to 300 °C (see Fig. 5a), a negative shift in the *V*_*T*_ is observed, which can be explained by the thermionic emission model (i.e., lesser energy is needed for electrons to overcome the Schottky barrier at higher temperatures). The RB-CRD is capable of outputting a steady regulating current up to 200 V (see Fig. 5b), which is higher than the reported maximum operation voltage of the Si-based commercial CRDs [22–24]. At 25 °C, the regulating current ratio (*I*_200 *V*_/*I*_25 *V*_) of the proposed RB-CRD is 0.998 indicating that the output current is quite steady. Thanks to the intrinsic high-temperature operation capability of AlGaN/GaN platform, the RB-CRD exhibits negligible degradation in the steadiness of the *I*_*A*_ up to 200 V at temperatures as high as 300 °C. Meanwhile, with the temperature increasing from 25 to 300 °C, the forward *I*_*A*_ reduces from 31.1 to 23.1 mA/mm due to the decreased electron mobility at elevated temperatures, as shown in Fig. 5b. The temperature coefficients (*α*) of the regulating current at different temperature ranges can be calculated by the following formula

$$ \alpha =\frac{I_1-{I}_0}{I_0\left({T}_1-{T}_0\right)}\times 100\% $$

where *I*_0_ is the output current at temperature *T*_0_ and *I*_1_ is the output current at temperature *T*_1_. A small temperature coefficient less than − 0.152%/^o^C is observed, indicating that the fabricated RB-CRD features excellent thermal stability.

**Fig. 6:**
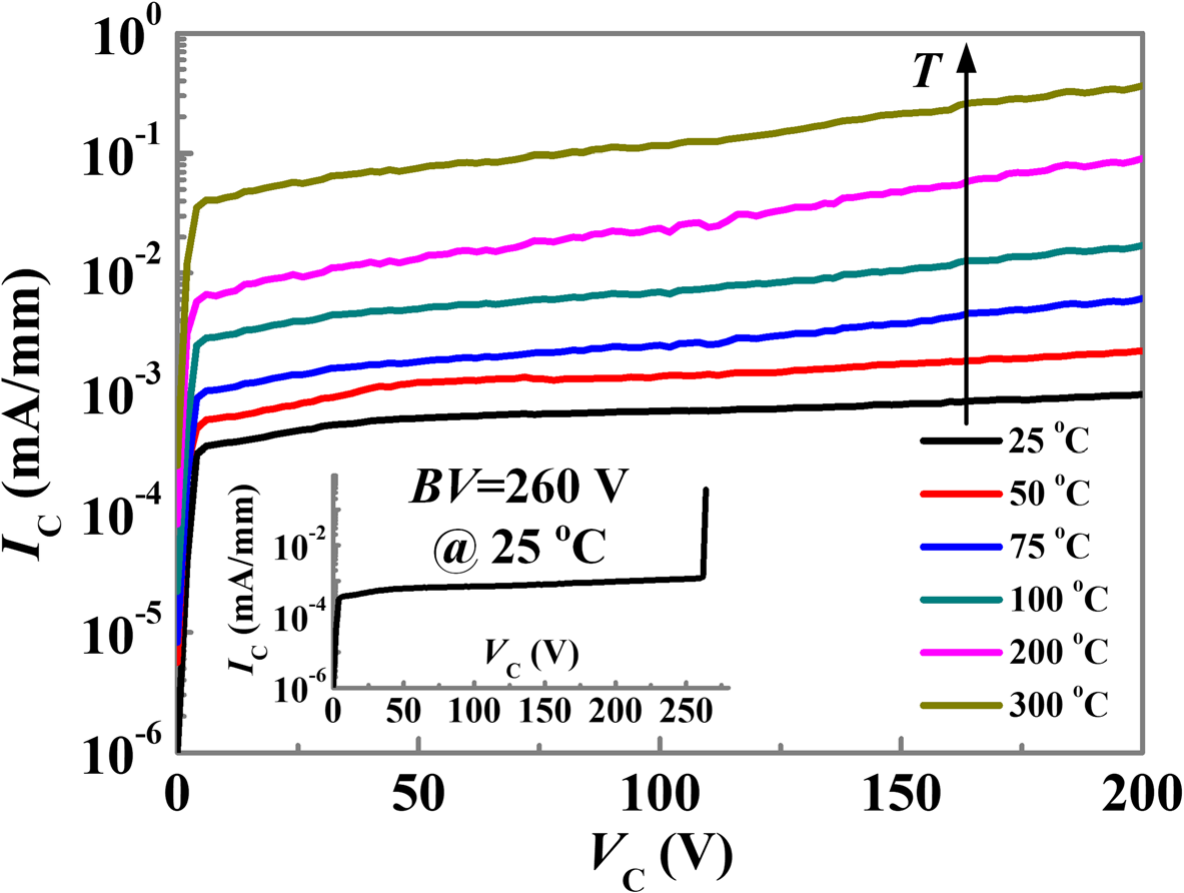
Temperature dependent reverse bias *I*-*V* characteristics of the RB-CRD

As shown in the inset of Fig. 6, the reverse breakdown voltage of the RB-CRD is 260 V at 25 °C. The corresponding average critical electric field is calculated to be 0.65 MV/cm. The temperature dependent reverse *I-V* characteristics of the RB-CRD are shown in Fig. 6. The increase of the ambient temperature from 25 to 300 °C gives rise to an increase of the leakage current by two orders of magnitude.

## Conclusions

In conclusion, a novel AlGaN/GaN-on-Si RB-CRD featuring trench Schottky anode and hybrid trench cathode has been successfully demonstrated for the first time. The fabricated RB-CRD exhibits a *V*_*K*_ of 1.3 V, a forward operation voltage over 200 V, and a reverse breakdown voltage of 260 V. An excellent accuracy as well as small negative temperature coefficient less than − 0.152%/^o^C have been obtained for the RB-CRD. The multifunctional RB-CRD with high accuracy is of great potential to be incorporated into emerging GaN power electronics systems.

## Abbreviations

2DEG: Two-dimensional electron gas
AFM: Atomic force microscope
ICP: Inductively coupled plasma
MOCVD: Metal organic chemical vapor deposition
RB-CRD: Reverse blocking current regulating diode
SBD: Schottky barrier diode
